# Prediction and characterization of novel epitopes of serotype A foot-and-mouth disease viruses circulating in East Africa using site-directed mutagenesis

**DOI:** 10.1099/vir.0.000051

**Published:** 2015-05

**Authors:** Fufa Dawo Bari, Satya Parida, Amin S. Asfor, Daniel T. Haydon, Richard Reeve, David J. Paton, Mana Mahapatra

**Affiliations:** ^1^​ The Pirbright Institute, Ash Road, Woking, Surrey, GU24 0NF, UK; ^2^​ Boyd Orr Centre for Population and Ecosystem Health, Institute of Biodiversity, Animal Health and Comparative Medicine, College of Medical, Veterinary and Life Sciences, University of Glasgow, G12 8QQ, UK

## Abstract

Epitopes on the surface of the foot-and-mouth disease virus (FMDV) capsid have been identified by monoclonal antibody (mAb) escape mutant studies leading to the designation of four antigenic sites in serotype A FMDV. Previous work focused on viruses isolated mainly from Asia, Europe and Latin America. In this study we report on the prediction of epitopes in African serotype A FMDVs and testing of selected epitopes using reverse genetics. Twenty-four capsid amino acid residues were predicted to be of antigenic significance by analysing the capsid sequences (*n* = 56) using *in silico* methods, and six residues by correlating capsid sequence with serum–virus neutralization data. The predicted residues were distributed on the surface-exposed capsid regions, VP1–VP3. The significance of residue changes at eight of the predicted epitopes was tested by site-directed mutagenesis using a cDNA clone resulting in the generation of 12 mutant viruses involving seven sites. The effect of the amino acid substitutions on the antigenic nature of the virus was assessed by virus neutralization (VN) test. Mutations at four different positions, namely VP1-43, VP1-45, VP2-191 and VP3-132, led to significant reduction in VN titre (*P* value = 0.05, 0.05, 0.001 and 0.05, respectively). This is the first time, to our knowledge, that the antigenic regions encompassing amino acids VP1-43 to -45 (equivalent to antigenic site 3 in serotype O), VP2-191 and VP3-132 have been predicted as epitopes and evaluated serologically for serotype A FMDVs. This identifies novel capsid epitopes of recently circulating serotype A FMDVs in East Africa.

## Introduction

Foot-and-mouth disease (FMD) is a highly infectious, rapidly spreading and internationally important livestock disease. It has significant socio-economic consequences due to losses in production and constraints on export of live animals and associated products to disease-free countries. FMD is caused by FMD virus (FMDV) that belongs to the family *Picornaviridae*, genus *Aphthovirus*. The virus exists as seven distinct serotypes (A, O, C, Asia1, South African Territory (SAT)-1, SAT-2 and SAT-3) that differ genetically and antigenically with multiple strains in different continents. Globally, most outbreaks of FMD are caused by serotype O followed in frequency by serotype A (Rweyemamu *et al.*, 2008; Chitray *et al.*, 2014; Wekesa *et al.*, 2014), which is endemic in many developing countries of Africa and Asia. FMDV serotype A continues to cause outbreaks in East Africa (Bari *et al.*, 2014; Wekesa *et al.*, 2014). It is a small, non-enveloped virus containing a single-stranded positive-sense RNA genome. The genome has a single ORF that encodes four capsid (structural) proteins and 10 non-structural proteins (Grubman & Baxt, 2004). As an RNA virus, it is characterized by the frequent emergence of new variants responsible for recurring disease outbreaks.

The genetic heterogeneity of the FMDV arises from lack of proofreading mechanisms during virus replication resulting in new variants, including those with changes in antigenically important sites of the virus (VP1-3) that may improve viral fitness. These sites are commonly investigated *in vitro* by epitope mapping using mAb (Thomas *et al.*, 1988; Bolwell *et al.*, 1989; Kitson *et al.*, 1990; Crowther *et al.*, 1993; Mateu *et al.*, 1995; Mahapatra *et al.*, 2011; Grazioli *et al.*, 2013). Four antigenic sites (equivalent to sites 1, 2, 4 and 5 of serotype O) were described for serotype A; site 1 (G-H loop of VP1) is linear and trypsin-sensitive, whereas the others are conformational and trypsin-resistant (Thomas *et al.*, 1988; Bolwell *et al.*, 1989; Saiz *et al.*, 1991; Mahapatra *et al.*, 2011). Escape mutants are also studied using polyclonal antibodies in serotypes O and C (Rojas *et al.*, 1992; Schiappacassi *et al.*, 1995; Kumar *et al.*, 2004; Sarangi *et al.*, 2013). In addition, the location of antibody binding sites (epitopes) can be inferred from correlating the antibody cross-reactivity of viruses to their capsid sequence similarities (Reeve *et al.*, 2010).

Epitopes can also be predicted from three-dimensional (3D) structural data alone, from aligned sequence data alone, or by using both sequence data and three-dimensional structural data. More than 90 % of B-cell epitopes are conformational (Barlow *et al.*, 1986), where distantly located residues on a sequence come together during protein folding, and prediction of these epitopes is better performed by combining structural and sequence information. Various structure-based epitope prediction programs are available freely (Rubinstein *et al.*, 2009; Kringelum *et al.*, 2012; Qi *et al.*, 2014). DiscoTope (Kringelum *et al.*, 2012), for example, uses a combination of statistical difference in amino acid composition between epitope and non-epitope residues, structural proximity and physico-chemical properties of neighbourhood amino acids, and a surface measure. All structure-based methods retrieve data from the protein database (PDB) file and conduct blast searches for closely related sequences. DiscoTope, Epitopia and SEPPA were recently applied to FMDV epitope prediction by Borley *et al.* (2013).

The importance of predicted residues for antibody binding can be tested by introducing specific mutations into a cDNA clone of the virus of interest. This approach is widely applied in emerging virus investigations including those into influenza (Yang *et al.*, 2013), FMDV (Blignaut *et al.*, 2011; Asfor *et al.*, 2014; Opperman *et al.*, 2014) and human immunodeficiency virus type 1 (HIV-1) (Evans *et al.*, 2014).

Epitopes of many FMDV serotype A strains originating from Asia, Europe and Latin America are well characterized using monoclonal antibody resistant (mar) studies (Thomas *et al.*, 1988; Baxt *et al.*, 1989; Saiz *et al.*, 1991; Mahapatra *et al.*, 2011). However, there are no reports for analysis of epitopes using serotype A isolates originating from East Africa. In this study, we studied viruses from East Africa and report prediction of epitopes, including amino acid residues not reported previously for serotype A viruses. Eight of the predicted epitopes were tested using a cDNA clone and their antigenic impact was assessed by virus neutralization (VN) test, revealing neutralizing epitopes at positions VP1-43, -45, VP2-191 and VP3-132.

## Results and Discussion

Antibodies play an important role in conferring protection against FMDV, including the protective effect of vaccination (Pay and Hingley, 1987; McCahon *et al.*, 1989), which is derived from antibodies directed towards the surface of the inactivated capsids. Identification of the epitopes and understanding their immunodominance in antigenically and genetically diverse FMDVs is of utmost importance for vaccine strain selection and novel vaccine development to achieve adequate protection against the disease (Doel, 1996; Paton *et al.*, 2005; Parida, 2009).

### Epitope prediction

Studies on the critical amino acid residues for neutralization of serotype A FMDV have used mainly mar-mutant approaches with viruses from the Middle East (A22) (Bolwell *et al.*, 1989), India (A/IND/17/77) (Tosh *et al.*, 1999), Europe [A5 (Saiz *et al.*, 1991), A10 (Thomas *et al.*, 1988), A12 (Baxt *et al.*, 1989)] and South America (A24) (Mahapatra *et al.*, 2011). Consequently, there is no information available on the epitopes present on the serotype A viruses isolated from Africa. Therefore, as an initial step, we analysed a collection of East African serotype A viruses (i) by using two freely available software programs to predict epitopes from capsid amino acid sequences with and without structural information [in this case, the A10_61_ PDB file (1ZBE)] and (ii) by correlating differences in neutralizing serum titres between virus pairs with capsid amino acid sequence changes.

#### Epitopes predicted by *in silico* methods.

The results of Shannon entropy and ConSurf analysis are presented in Table 1. High Shannon entropy signifies amino acid variability and high values have been reported for variable epitopes in HIV (Liu *et al.*, 2013; Evans *et al.*, 2014), influenza (Pan & Deem, 2011) and neutralization escape FMD viruses (Piatti *et al.*, 1995; Maree *et al.*, 2011). In Shannon entropy analysis, use of a threshold of 0.86 (half of the highest score) resulted in the selection of 33 candidate amino acid residues to be of antigenic significance.

**Table 1.** List of capsid amino acid residues and their scores from entropy and ConSurf analysis Amino acids are arranged according to their order in the P1 sequence. The predicted amino acid residues are mostly located close to known epitopes except for residue VP2-191 that is 5 aa apart from VP2-196. nr, No reference antigenic site reported; GHL, VP1 G-H loop.

P1 position	Viral protein (VP)	VP position	Entropy value	ConSurf score	Epitope reported previously	Residue located on external surface
159	2	74	1.1	3.985	nr	Yes
163	2	78	1.156	4.458	nr	Yes
171	2	86	1.183	4.472	nr	Yes
173	2	88	0.867	3.644	nr	Yes
218	2	133	1.37	4.662	nr	Yes
219	2	134	1.704	4.802	134	Yes
276	2	191*	1.627	4.789	nr	Yes
292	2	207	1.018	3.65	nr	No
338	3	35	0.988	3.523	nr	No
373	3	70	1.031	4.541	70	Yes
374	3	71	1.348	5.29	71	Yes
434	3	131*	0.931	3.074	nr	Yes
523	3	220*	1.25	5.034	nr	Yes
566	1	42	1.096	2.529	nr	Yes
567	1	43*	1.222	2.996	nr	Yes
568	1	44*	1.42	3.555	nr	Yes
569	1	45*	1.554	3.861	nr	Yes
623	1	99	1.585	4.287	nr	Yes
625	1	101	1.078	2.38	nr	Yes
663	1	139	1.177	2.855	139	GHL
666	1	142	1.185	2.915	142	GHL
673	1	149	1.263	3.132	149	GHL
721	1	197	1.484	3.783	nr	Yes
722	1	198	1.343	3.3	198	Yes

* Residues selected for site-directed mutagenesis study.

**Fig. 1.**
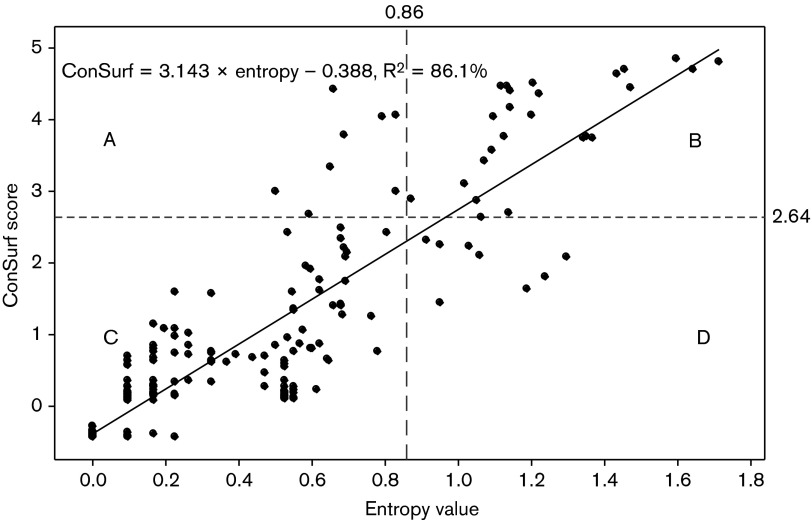
Scatter plot of Shannon entropy and ConSurf values showing areas of concordant high values (top right box, B) corresponding to the 24 commonly predicted amino acids. The cut-off values are indicated by black dotted lines. The high R^2^ value (86.1 %) indicates good correlation of the two prediction results. The graph was drawn using Minitab V.16 statistical software.

**Fig. 2.**
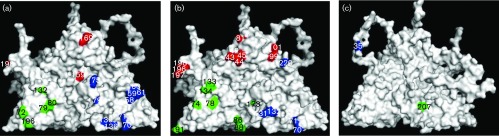
A10_61_ (1ZBE) structure showing (a) the critical residues of reported epitopes; epitopes predicted in this study (b) external surface, (c) internal surface. Red, green and blue colours indicate residues in VP1, VP2 and VP3, respectively.

The highest antigenicity score in ConSurf was 5.29 and the top scoring 33 amino acid positions were compared with the entropy prediction results. Out of these, 24 were selected by both methods showing good agreement (86 %) between the two prediction methods (Fig. 1). All the 24 predicted residues were located on the outer surface of the virus capsid (Fig. 2b) except two residues, VP2-207 and VP3-35, which were internal (Fig. 2c). Eleven, eight and five predicted residues are present in VP1, VP2 and VP3, respectively (Table 1). Out of these, seven (29.2 %) have been previously reported in serotype A viruses; VP1-139/141/142, -149 (Thomas *et al.*, 1988), VP1-198 (Saiz *et al.*, 1991), VP3-70 (Thomas *et al.*, 1988) and VP2-134 were reported to be of antigenic significance in serotype A viruses previously (Saiz *et al.*, 1991) or to influence mAb binding in serotype O (Mahapatra *et al.*, 2008). Previously, *in silico* epitope predictions performed using the A10_61_ crystal structure identified six (VP1-196/197/198, VP2-191 and VP3-70/71) of the 24 residues (Borley *et al.*, 2013). Because ConSurf predicts epitopes with reference to the 3D structure and also by comparing evolutionary conservation rates of the aligned amino acid sequences, it is expected that it might provide more specific predictions of epitopes than would entropy analysis. Accordingly, ConSurf selected VP1-148 (data not shown), a neutralizing conformational epitope reported earlier for serotype A (Mahapatra *et al.*, 2011).

This is the first report, to our knowledge, to predict that residues at VP1-43/44/45 could have potential antigenic significance in serotype A FMDV. In addition, the neighbouring residue VP1-46 is also highly variable and together they form a cluster of residues on the capsid surface. Many of the previously identified regions of high amino acid variability on the P1 sequence of serotypes A and O FMDVs correspond to known antigenic sites and these positions are conserved structurally between the two serotypes (Fry *et al.*, 2005; Chitray *et al.*, 2014). This region corresponds to antigenic site 3 in serotype O (Kitson *et al.*, 1990). In serotype O the stability of antigenic site 3 has been considered important for the stability of the VP1 G-H loop, and any destabilization in VP1 residues 43 to 45 may distort the conformation of the flexible VP1 G-H loop (Fry *et al.*, 2005). Opperman *et al.* (2014) also recently reported the binding of monoclonal antibodies to closely located residues VP1-48 to -50 in the SAT2 serotype of FMDV. In addition, both ConSurf and entropy analysis predicted VP1-99 and -101 to be of antigenic significance whilst VP1-110 was predicted by entropy analysis only. A recent study in SAT2 FMDVs also suggested the presence of epitopes at VP1-109 and -111 (Opperman *et al.*, 2014). In VP2, of the eight residues predicted by both methods only two (VP2-134 and VP2-191) were indicated to be of antigenic significance. The remaining six residues were newly predicted (Table 1) *in silico* to be of antigenic importance but their relevance so far could not be confirmed by other methods. The amino acid at position VP2-191 is located at the threefold axis of the capsid and is among the top four amino acids predicted by both *in silico* methods. This residue has been recently reported to be a neutralizing epitope linked to antigenic site 2 in serotype O FMDV (Asfor *et al.*, 2014). In VP3, a total of five residues were predicted of which three (35, 71 and 131) were newly predicted. VP3-70 was previously reported by mar-mutant studies (Thomas *et al.*, 1988). Recently, VP3-220 has been indicated to be of antigenic significance in serotype A viruses (Upadhyaya *et al.*, 2014) and is located close to other newly predicted residues (VP1-99 and VP1-101) on the outer surface of the capsid (Fig. 2b).

#### Epitopes predicted by correlating sequence and serology data.

A total of six residues were predicted as epitopes by correlating serum titres and changes in capsid amino acid sequences, namely residues VP1-81, -138, -148 and -159, VP2-79 and VP3-132. Of these, four residues, VP1-138, -159 (Thomas *et al.*, 1988), VP1-148 (Mahapatra *et al.*, 2011) and VP2-79 (Saiz *et al.* 1991), were reported previously using mar-mutant studies or are within the VP1 G-H loop. Though VP3-135 has been reported by mar-mutant studies in SAT1 virus (Grazioli *et al.*, 2006), residues VP1-81 and VP3-132 have not been reported previously in serotype A viruses and were good candidates for further investigation using a cDNA clone.

Among all the epitopes predicted by the *in silico* methods, residue VP2-191 was among the top four predicted epitopes and has not been reported previously by mar-mutant studies. VP1-43, -44 and -45, equivalent to antigen site 3 in serotype O virus, was predicted by both the *in silico* methods and was therefore selected for further investigation. In addition, the epitopes at VP1-81 and VP3-132 uniquely predicted by correlating sequence and serology data were taken forward for further investigation. VP3-131 predicted by ConSurf is located next to VP3-132 on the external surface and was taken forward for further investigation. VP3-220 predicted by both the *in silico* methods was also selected for further investigation.

### Generation of full-length genome plasmids

The capsid-coding region of serotype A FMDV (A-EA-2007) was cloned successfully into the plasmid pT7S3-O1Kwt to generate the full-length genome plasmid pT7S3/A-EA-2007. This plasmid was used as the template to introduce further mutations in the capsid-coding region. A total of eight residues (VP1-43, -44, -45, -81, VP2-191 and VP3-131, -132, -220) were selected for this purpose as they were indicated to have an impact on the antigenicity of the virus by comparison of capsid sequences with *in vitro* virus cross-neutralization data or by epitope prediction using capsid sequence and viral crystal structure, and were novel (not reported previously). A total of 12 single mutant plasmids involving seven residues were generated (Table 2). The capsid coding regions of all the plasmids were sequenced on both strands and no unwanted mutations were observed.

**Table 2.** List of O1K/A-EA-2007 mutant viruses generated in this study and their associated amino acid substitutions Positions different from rO1K/A-EA-2007 are shaded.

Virus	Capsid amino acid substitutions						
VP1			VP2	VP3		
43	44	45	191	131	132	220
rO1K/A-EA-2007	N	S	L	T	E	T	Q
rO1K/A-EA-2007M1	A	S	L	T	E	T	Q
rO1K/A-EA-2007M2	N	A	L	T	E	T	Q
rO1K/A-EA-2007M3	N	P	L	T	E	T	Q
rO1K/A-EA-2007M4	N	S	P	T	E	T	Q
rO1K/A-EA-2007M5	N	S	L	A	E	T	Q
rO1K/A-EA-2007M6	N	S	L	D	E	T	Q
rO1K/A-EA-2007M7	N	S	L	T	A	T	Q
rO1K/A-EA-2007M8	N	S	L	T	D	T	Q
rO1K/A-EA-2007M9	N	S	L	T	E	A	Q
rO1K/A-EA-2007M10	N	S	L	T	E	S	Q
rO1K/A-EA-2007M11	N	S	L	T	E	T	A
rO1K/A-EA-2007M12	N	S	L	T	E	T	T

### Rescue and characterization of recombinant viruses from full-length genome plasmids

Live infectious viruses were recovered successfully from all the cDNA clones following electroporation. FMDV-specific cytopathic effect (CPE) was observed 18–24 h post-electroporation. Extensive CPE was observed at both the first and second passages. At least two independent clones of each virus were rescued. However, only one clone in each case was used for further characterization. In order to establish that the expected viruses had been rescued, reverse transcription (RT)-PCR was carried out on the RNA extracted from infected BHK-21 cells using primer pair L460F and EUR2B52R/NK72 that produced a 2500 bp fragment (encompassing C-terminal part of L, P1, 2A and N-terminal part of 2B) of expected size (data not shown). No PCR products were generated in parallel reactions in which the enzyme reverse transcriptase was omitted, indicating that the products amplified were not generated from the transfected plasmid DNA. The PCR products were sequenced on both the strands and no additional nucleotide substitutions were observed in any of the mutants generated in this study.

The parent virus A-EA-2007 represents genotype VII of serotype A FMDV circulating in East Africa. Previously, chimeric viruses containing capsid-coding regions derived from serotype A/Turkey 2/2006 or O/UKG/34/2001 FMDV with the backbone of serotype O1K cDNA clone (pT7S3-O1K) have been generated successfully (Bøtner *et al.*, 2011). These chimeric viruses retained the characteristics (in terms of receptor utilization, antigenicity, pathogenicity, etc.) from the parent from which the capsid was derived. In addition, successful switching of capsids from other serotypes of FMDV for the purpose of recombinant vaccine development and evaluation has been reported (van Rensburg *et al.*, 2004; Blignaut *et al.*, 2011; Zheng *et al.*, 2013).

The mutants were stable genetically at least up to third passage as confirmed by full capsid sequencing. Standard multi-step growth curves were carried out to compare the growth of the recombinant viruses with that of the parent virus. All the viruses grew at a similar rate and to a similar titre, indicating the mutations in the antigenic sites had no adverse effects on the replication efficiency of these viruses *in vitro* (Fig. S1, available in the online Supplementary Material). The ability of FMDVs to tolerate changes at these positions is consistent with the observation of high amino acid variability at these residue positions in the 115 field viruses analysed [56 sequences reported before those of Bari *et al.* (2014) and the remaining 59 sequences downloaded from GenBank; data not shown]. BHK-21 cells infected with the parent or recombinant viruses were stained following infection and photographed. Both the parent and the recombinant viruses exhibited variable size plaques with no clear differences between them (data not shown). This corroborates the findings in a recent study of serotype O FMD mutant viruses (Seago *et al.*, 2012; Lawrence *et al.*, 2013; Asfor *et al.*, 2014).

### Serological reactivity of rO1K/A-EA-2007 mutant viruses

**Fig. 3.**
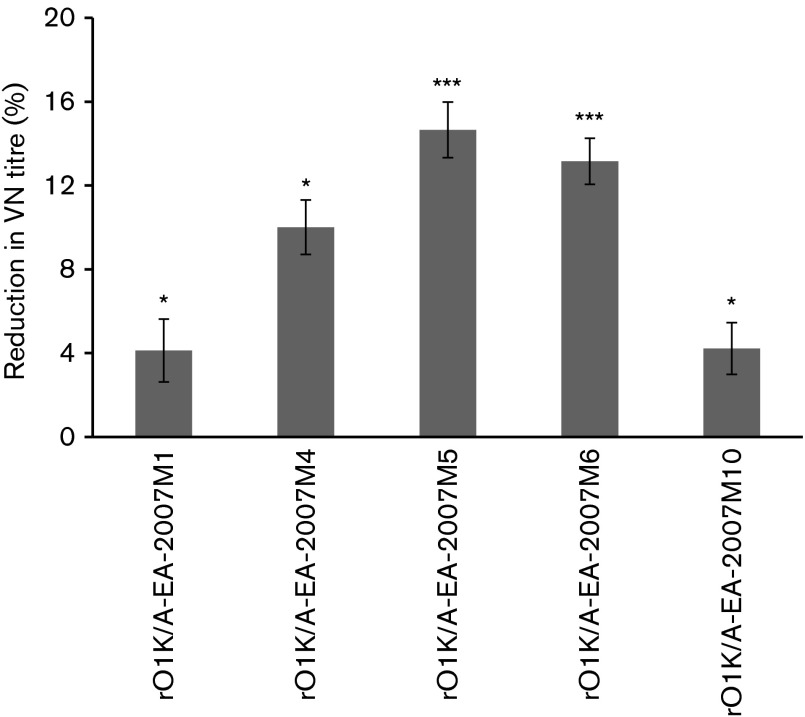
Percentage reduction in serum titre of selected rO1K/A-EA-2007 mutant viruses that showed significant reduction in serum titre compared with the parent rO1K/A-EA-2007 virus. 2D-VNT was carried out using bovine serum raised against rO1K/A-EA-2007 viral antigen. Error bars indicate sd for the respective mutant. The significance of the test differences were calculated from a total of 16 repeats except for rO1K/A-EA-2007M6 for which only 12 VN test results were used. * and *** indicate significant difference (from parent rO1K/A-EA-2007 virus) at *P*<0.05 and *P*<0.001, respectively.

The impact of the amino acid substitutions on sero-reactivity was assessed by VN test using the pooled post-vaccination serum (bovine) raised against rO1K/A-EA-2007 antigen. The main goal was to quantify the reduction in neutralization following mutations in the capsid of FMDV. Therefore, it was crucial to determine the VN titre of the sera against all the mutant viruses at a fixed virus dose (100 TCID_50_). Therefore, a two-dimensional micro-neutralization test (2D-VNT) was carried out using five different doses of the virus encompassing 100 TCID_50_ for this purpose. The resultant VN titres at each virus dose were used to calculate the serum titre at 100 TCID_50_ by regression analysis. Because getting consistent results was very important for the evaluation of the mutant viruses, each test was conducted in duplicate and repeated at least eight times. Test results showing evidence of a reduction in serum titre after mutagenesis were repeated eight more times for further confirmation. Among the 12 mutants generated in this study, only five mutants, i.e. rO1K/A-EA-2007M1 (VP1-L43A), rO1K/A-EA-2007M4 (VP1-L45P), rO1K/A-EA-2007M5 (VP2-T191A), rO1K/A-EA-2007M6 (VP2-T191D) and rO1K/A-EA-2007M10 (VP3-T132S), exhibited significant reductions in serum titre (Fig. 3). The substitution of threonine at VP2-191 to alanine or aspartic acid exhibited relatively greater (15 % and 12.5 %, respectively) reductions in serum titre as compared with the parent virus. This agrees with the report of Crowther *et al.* (1993) who reported ~15 % reduction in serum titre as a result of a single amino acid change. In line with this, recently, Asfor *et al.* (2014) evaluated this epitope for serotype O FMDV using a cDNA clone and reported ~ 30 % reduction in serum titre. Hence this residue could represent a novel epitope across several serotypes. The residues VP1-43 to -45 are in an equivalent position to antigenic site 3 in serotype O (Kitson *et al.*, 1990). Though mar-mutant studies have been carried out in several type A viruses, this region has never been reported to be of antigenic significance. However, VP1-45 has been indicated to impact on the antigenic nature of the serotype A viruses from the Middle East (Jamal *et al.*, 2011; Upadhyaya *et al.*, 2014). In addition, analysis of 115 serotype A capsid sequences revealed amino acids VP1-42 to -46 to be highly variable (data not shown). The substitution of threonine at position VP3-132 led to significant reduction in serum titre whereas substitution to alanine did not have much impact, indicating certain residue changes are more powerful than others. Mutations in epitopes may also have the opposite effect, i.e. neutralizing titres may increase after mutation of capsid residues. In fact, Opperman *et al.* (2014) reported significantly higher VN titres in SAT epitope-replaced mutants that were related to higher avidity index. However, we did not observe significantly higher VN titre in the mutant viruses in this study. In our previous study on serotype O epitope mutants (Asfor *et al.*, 2014) we also did not observe higher VN titre than the homologus virus. This could be due to a different serotype or strain of the virus.

In conclusion, we have predicted epitopes for serotype A viruses from Africa and tested a new epitope/antigenic site (VP1-43/45) for serotype A FMDVs that is equivalent to antigenic site 3 in serotype O. Substitution of threonine (amino acid found in genotype IV viruses) at position VP2-191 with either alanine or aspartic acid confirmed the antigenic significance of this residue as well as mutations at VP1-43, VP1-45 and VP3-132. These residues are novel epitopes that have not been reported previously for serotype A FMDVs.

## Methods

### Viruses, cells and plasmid.

The 56 East African type A viruses and their corresponding capsid sequences and serology data against seven vaccine strains described previously (Bari *et al.*, 2014) were used in this study. IB-RS2 cells (a pig kidney cell line) were used for growing viruses, titration and VN test. BHK21 cells were used for electroporation and passaging of recombinant viruses.

The most broadly reactive vaccine strain, A-EA-2007 (Bari *et al.*, 2014), was selected for the reverse genetics work in this study. The parental virus was plaque-purified four times on IB-RS2 cells. An existing serotype O infectious copy of FMDV (pT7S3-O1Kwt) containing unique restriction sites at the beginning and end of the capsid-encoding genes (*Afl*II and *Spe*I restriction sites in the L and 2B regions of the FMDV genome, respectively; Asfor *et al.*, 2014) was used for this study.

### RNA extraction, RT-PCR and sequencing.

Total RNA was extracted from cell-culture-grown viruses using RNeasy Mini kits (Qiagen) according to the manufacturer’s recommendation. Reverse transcription (RT)-PCR to amplify the capsid-coding region, nucleotide sequencing and sequence analysis were carried out as described by Upadhyaya *et al.* (2014).

### Construction of recombinant full-length genome plasmid.

Standard molecular biological techniques were used for the cloning of the serotype A capsid into vectors. The A-EA-2007 FMDV capsid-encoding region was amplified from the total RNA isolated from the plaque-purified virus using specific primer set A-EA-2007 *Afl*II F/A-EA-2007*Spe*I R containing *Afl*II and *Spe*I restriction sites, respectively (Table S1). The amplified product was cloned in to the intermediate vector pT7Blue (Promega) resulting in generation of pT7Blue-A-EA-2007. The capsid was excised from the intermediate vector using *Afl*II and *Spe*I restriction enzymes. The excised capsid-encoding region was used to replace the capsid-encoding region of pT7S3-O1Kwt to generate pT7S3-O1K/A-EA-2007. The capsid-encoding region of the full-length genome plasmid was sequenced on both the strands to ensure they were from the desired virus. pT7S3-O1K/A-EA-2007 was used in all subsequent experiments.

#### Epitope prediction.

##### *In silico* methods.

Two different methods of epitope prediction, (a) Shannon entropy (Shannon, 1948) and (b) conservation surface mapping (ConSurf) (Landau *et al.*, 2005; Ashkenazy *et al.* 2010), were used to predict candidate epitopes from the capsid sequence data of 56 serotype A FMDVs originating from Africa (Bari *et al.*, 2014). The Shannon entropy analysis implemented in BioEdit v7.2.5 (Hall, 1999) was used to calculate the variability of each amino acid position across all VP1-3 sequences taking account of the type and frequency of amino acids. In Shannon entropy, a score more than half of the highest score was used as a cut-off to select the most variable residue. In ConSurf (http://consurf.tau.il), the prediction of epitopes was performed by calculating the evolutionary conservation rate (inverse of evolutionary substitution rate) using a Bayesian method (Ashkenazy *et al.* 2010) for each position in the multiple amino acid sequence alignments of individual VP regions (VP1-3). The three-dimensional crystal structure of serotype A10_61_ FMDV (Fry *et al.*, 2005) was used as the reference structure. ConSurf also predicts the location of each amino acid (buried or surface exposed) and assigns an antigenicity score.

##### By correlating capsid sequence with serology data.

The locations of residues involved in antibody binding sites (epitopes) were inferred by correlating the antibody cross-reactivity of viruses to their capsid sequence similarities as described previously (Reeve *et al.*, 2010). This technique identifies residues responsible for cross-reactivity while controlling for repeated measures arising from the phylogenetic relationship between the viruses. The capsid sequence data of 56 East African type A viruses and their corresponding serology data against seven vaccine strains (Bari *et al.*, 2014) were used for this analysis.

### Construction of mutant plasmids.

Standard site-directed mutagenesis (SDM) technique was used to introduce mutations at specific positions in the capsid of pT7S3-O1K/A-EA-2007. A total of eight sites (VP1-43/44/45/81; VP2-191, VP3-131/132/220), all in surface-exposed capsid regions and newly predicted (not previously reported), were selected for further investigation. These residues were substituted either to alanine or with a specific amino acid whose presence in other isolates had been correlated with higher or lower antibody reactivity. Specific overlapping primers were designed to introduce mutations either individually or in combinations to make recombinant viruses containing either single or double substitutions in the capsid (Table S1). The cDNA backbone containing the serotype A capsid was manipulated according to the method described by Asfor *et al.* (2014). The capsid-encoding regions of all the plasmids were sequenced to confirm the identity of the mutations introduced.

### Electroporation and rescue of recombinant viruses.

The parent plasmid (pT7S3-O1K/A-EA-2007) or its derivatives were linearized by digestion with *Hpa*I enzyme and full-length RNA transcripts were synthesized as described previously (Asfor *et al.*, 2014). Electroporation of the transcribed RNA and recovery of the recombinant viruses were also carried out as described previously (Asfor *et al.*, 2014). The rescued viruses were subsequently passaged at least three times before stocks of viruses were made.

### Characterization of recombinant viruses.

In order to characterize the recombinant viruses, RT-PCR was carried out on the total RNA isolated from virus-infected BHK-21 cells. The whole capsid was amplified and then sequenced on both strands to ensure all the mutations were present in the respective viruses. Virus titres for each virus were determined on IB-RS2 cells as described by Reed & Muench (1938). The growth kinetics of the mutant and parental recombinant viruses were evaluated as described by Asfor *et al.* (2014). The plates were frozen at five different time points (0, 4, 8, 12 and 24 h) post-infection. The harvested virus was stored at −70 °C until used. The plaque sizes of the mutant and the parent recombinant viruses were also compared, as described by Asfor *et al.* (2014).

### Generation of polyclonal sera against rO1K/A-EA-2007 vaccine in cattle.

For use in serological assays antisera were prepared in cattle against the recombinant parent virus recovered from the cDNA clone (rO1K/A-EA-2007), which is the parent to all other mutant viruses generated in this study. Briefly, the rO1K/A-EA-2007 virus was grown in BHK-21 cells. When the CPE was complete, the culture supernatant was harvested, clarified by centrifugation, and inactivated with 5 mM binary ethylenimine (BEI) at 25 °C for 24 h (Bahnemann, 1975, 1990). The inactivated antigen was purified by sucrose gradient purification (Ferris *et al.*, 1984) and concentrated by polyethylene glycol precipitation. The vaccine was prepared from the antigen as a water-in-oil-in-water emulsion with Montanide ISA 206 (SEPPIC) adjuvant according to the manufacturer’s recommendation (1 : 1 ratio). Five cattle housed at the high-containment isolation facility of The Pirbright Institute, Pirbright, UK, were used for serum production. All the animals were sero-negative for FMDV antibodies at the beginning of the experiment. The animals were observed for 7 days before the beginning of the experiment to make sure that they were in good health. The animals were injected subcutaneously with 1 ml of an emulsion containing 15 µg each of the recombinant viral antigen. All the animals received a booster at 21 days post-vaccination and were bled 1 week later for serum preparation. The serum was stored at −20 °C until use. A pool of sera from five animals was used for serological tests.

### Virus neutralization test.

The 2D-VNT was carried out using the pooled 28th day post-vaccination bovine sera from five animals to determine the sero-reactivity of the mutant viruses according to Rweyemamu *et al.* (1978). The recombinant virus, rO1K/A-EA-2007, was used as the homologous virus in the VN test. Antibody titres were calculated from regression data as the log_10_ reciprocal antibody dilution required for 50 % neutralization of 100 tissue culture infective units of virus (log_10_SN_50_/100 TCID_50_). The antigenic relationship of a mutant virus to its parent is given by the ratio: ‘r_1_’ = neutralizing antibody titre against the mutant virus/neutralizing antibody titre against the recombinant parental virus. The significance of differences between ‘r_1_-values’ obtained by the polyclonal serum was evaluated according to the method of Rweyemamu & Hingley (1984) using a cut-off r_1_-value ≥0.3 as representing an expectation of adequate cross protection. Each test was conducted in duplicate and repeated at least eight times. Test results showing evidence of a reduction in serum titre after mutagenesis were repeated eight more times for further confirmation.

### Data analysis.

The data were analysed using minitab (version 16) software. A paired *t*-test was used to compare the differences in sero-reactivity between the homologous and the mutant viruses.
